# The relationship between maternal psychopathology and offspring incontinence and constipation at school age: A prospective cohort study

**DOI:** 10.1016/j.jad.2023.05.003

**Published:** 2023-05-07

**Authors:** Gemma Sawyer, Jon Heron, Carol Joinson

**Affiliations:** a MRC Integrative Epidemiology Unit, University of Bristol, Bristol, UK; b Population Health Sciences, Bristol Medical School, University of Bristol, Bristol, UK; c Centre for Academic Mental Health, Population Health Sciences, Bristol Medical School, University of Bristol, Bristol, UK; d Centre for Academic Child Health, Population Health Sciences, Bristol Medical School, University of Bristol, Bristol, UK

**Keywords:** Maternal psychopathology, Childhood incontinence, Constipation, Cohort study, Avon Longitudinal Study of Parents, Children (ALSPAC)

## Abstract

**Background:**

There is evidence for an association between maternal psychopathology and offspring incontinence and constipation, but it is unclear if there is a critical/sensitive period of exposure to maternal depression and/or anxiety in the antenatal or postnatal period.

**Methods:**

Mothers from the Avon Longitudinal Study of Parents and Children provided data on their depression and anxiety (antenatal and postnatal) and their child’s urinary and faecal incontinence and constipation at age 7 (6489 participants). We used multivariable logistic regression to examine evidence for independent effects of maternal depression/anxiety on offspring incontinence/constipation and to investigate if there was a critical/sensitive period of exposure. We examined evidence for causal intra-uterine effects using a negative control design.

**Results:**

Postnatal maternal psychopathology was associated with an increased risk of offspring incontinence and constipation (e.g. postnatal anxiety and daytime wetting OR: 1.53; 95 % CI: 1.21−1.94). Data were consistent with a postnatal critical period model and there was evidence for an independent effect of maternal anxiety. Antenatal maternal psychopathology was associated with offspring constipation (e.g. antenatal anxiety OR: 1.57; 95 % CI: 1.25−1.98), but there was no evidence for a causal intra-uterine effect.

**Limitations:**

Attrition and maternal reports without use of diagnostic criteria for incontinence/constipation are potential limitations.

**Conclusions:**

Children exposed to maternal postnatal psychopathology had a greater risk of incontinence/constipation, and maternal anxiety had stronger associations than depression. Health professionals should be vigilant to effects of maternal psychopathology on child development. Identification of mechanisms linking maternal psychopathology to child incontinence/constipation is required to inform evidence-based support.

## Abbreviations

ALSPAC: Avon Longitudinal Study of Parents and Children
CCEI: Crown Crisp Experiential Index
EPDS: Edinburgh Postnatal Depression Scale
HPA: Hypothalamic-Pituitary-Adrenal
LR: Likelihood Ratio

## 1 Introduction

Maternal psychopathology has been identified as a key risk factor that is associated with adverse effects on multiple child outcomes including cognitive and emotional development (Beck, 1998), social engagement and stress reactivity (Feldman et al., 2009), internalising and externalising behaviour (Goodman et al., 2011), infant growth (Rahman et al., 2004), and physical health during childhood (National Research Council, 2009; Raposa et al., 2014). There is also some evidence that exposure to maternal psychopathology is associated with an increased risk of childhood incontinence and constipation, which are among the most common paediatric health problems. Most cases of paediatric incontinence arise from functional impairments in the bladder and/or bowel rather than organic causes. Daytime urinary incontinence and bedwetting (enuresis) are estimated to affect around 8 % and 15 % of 7-year-olds respectively (Butler et al., 2005; Joinson et al., 2006b). Soiling (encopresis) is also common, with around 6.8 % of children affected at age 7 (including 1.4 % who soil once a week or more) (Joinson et al., 2006a). Over 80 % of cases of childhood soiling are a consequence of chronic constipation, whilst around 20 % have no underlying constipation (non-retentive) (Bongers et al., 2007). Despite the common belief that incontinence resolves with age, it can persist into adolescence, with prevalence estimates of around 3 % for daytime wetting and 2.5 % for bedwetting (Heron et al., 2017). Moreover, among children with soiling, 15 % are estimated to continue to be affected at age 18, whilst 20 % with constipation continue to experience symptoms at age 16 (Bongers et al., 2007). Incontinence has adverse consequences for children and adolescents, including low self-esteem, difficult peer relationships, and depressive symptoms (Grzeda et al., 2017; Thibodeau et al., 2013). It is, therefore, important to identify modifiable risk factors, possibly including maternal psychopathology, that could increase the risk of children having problems attaining and maintaining continence.

Cross-sectional studies have reported higher levels of psychopathology in mothers of children with bedwetting (Durmaz et al., 2017), constipation (Appak et al., 2017), and soiling (Akdemir et al., 2015) compared to mothers of children without these problems. Clinical studies have found higher anxiety scores in mothers of children with enuresis compared to controls without incontinence (Naitoh et al., 2012). These studies are unable to disentangle the direction of association between maternal psychopathology and childhood incontinence because reverse causality is plausible, e.g. mothers of children with bedwetting had decreased state anxiety following their child’s successful treatment (Naitoh et al., 2012). In addition, these studies failed to adequately adjust for confounders.

A prospective study found that lifetime parental psychopathology is associated with daytime wetting and bedwetting in offspring but did not adjust for confounders (Kessel et al., 2017). Other prospective studies have found that maternal psychopathology in the antenatal and postnatal periods is associated with children’s incontinence up to age 9 (Joinson et al., 2019a, 2009, 2008). These studies leave several questions unanswered. Firstly, they did not examine whether maternal depression and anxiety have independent effects on offspring incontinence/constipation. Although maternal anxiety and depression are highly comorbid, they may have different effects on offspring development (Glover, 2014). Secondly, it is unclear whether exposure to maternal psychopathology in the antenatal or postnatal period has a stronger association with the risk of offspring incontinence/constipation. According to the life course approach, adverse exposures during early childhood can have enduring influences on health and development (Moss et al., 2020). The sensitive period model hypothesises that exposure during a particular period increases the risk of an adverse outcome to a greater extent than exposure during another period, whereas the critical period model hypothesises that the risk of an adverse outcome is increased only when there is exposure occurs during a particular period (Moss et al., 2020). Thirdly, it is unclear whether intra-uterine exposure to maternal psychopathology has a causal effect on offspring incontinence/constipation. Mother’s partner’s exposures assessed during pregnancy can be used as negative controls in observational studies to improve causal inference regarding effects of antenatal exposures on offspring because they have similar confounding structures to maternal antenatal exposures, but no plausible biological link with offspring outcomes. If maternal, but not partner, exposures are associated with offspring outcomes, this would provide evidence of a possible causal intra-uterine effect. It has been suggested that intrauterine exposure to maternal psychopathology is associated with alterations in the foetal Hypothalamic-Pituitary-Adrenal (HPA) axis, which can affect child development (Glover, 2014). Alternatively, if both mother and partner exposures are associated with offspring outcomes, confounding by shared genetic or environmental factors, such as family adversity and increased stress, are the more likely explanations (Lipsitch et al., 2010).

The current study uses data from a large UK birth cohort to examine the relationship between maternal psychopathology (depression and anxiety) and offspring incontinence (daytime wetting, bedwetting, soiling) and constipation at 7 years. The objectives are to examine (i) evidence for independent effects of maternal depression and anxiety on these offspring outcomes, (ii) whether there is a critical or sensitive period of exposure to maternal psychopathology in the antenatal or postnatal period, and (iii) evidence for a causal intra-uterine effect of maternal psychopathology on offspring incontinence and constipation with a negative control design.

## 2 Method

### 2.1 Study population

The Avon Longitudinal Study of Parents and Children (ALSPAC) is a UK population-based birth cohort which recruited pregnant women residing in the former Avon region with expected delivery dates between 1st April 1991 and 31st December 1992. The initial cohort included 14,541 pregnancies, resulting in 13,988 children who were alive at 1 year of age. Full details of the ALSPAC methods have been previously outlined (Boyd et al., 2013; Fraser et al., 2013). The study website contains details of available data through a fully searchable data dictionary and variable search tool (http://www.bristol.ac.uk/alspac/researchers/our-data/). Ethical approval for the study was obtained from the ALSPAC Ethics and Law Committee and the Local Research Ethics Committees. Informed consent for the use of data collected via questionnaires and clinics was obtained from participants following the recommendations of the ALSPAC Ethics and Law Committee at the time.

**Fig. 1:**
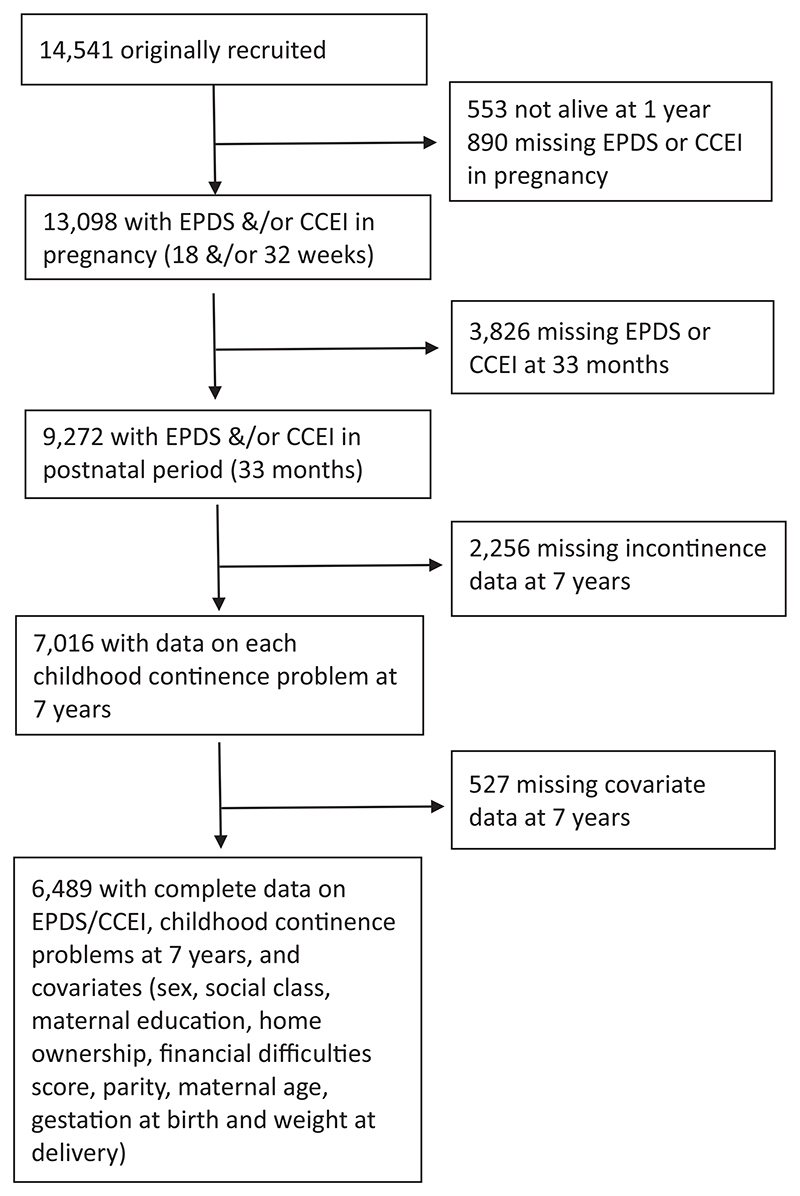
Flow chart showing the participants included in the study.

The current study uses a complete case sample of 6489 participants who provided data on all relevant variables (Fig. 1).

### 2.2 Exposures: maternal antenatal and postnatal depression and anxiety

At 18- and 32-weeks’ gestation and 33 months postnatal, mothers were invited to complete the Edinburgh Postnatal Depression Scale (EPDS), a validated measure for screening depression during and following pregnancy (Cox et al., 1987). Scores of 13 or higher are often used to indicate probable depression (Evans et al., 2001). We categorised mothers as ‘depressed’ or ‘not depressed’ in the antenatal and postnatal periods using this threshold. Mothers also completed the anxiety subscale of the Crown Crisp Experiential Index (CCEI) (Crisp et al., 1978). Higher scores indicate more severe anxiety and scores of 9 or higher have previously been used to indicate more severe anxiety (Joinson et al., 2009). We dichotomised mothers into ‘anxious’ and ‘not anxious’ using this threshold. We used the mean of EPDS or CCEI scores at 18- and 32-weeks’ gestation to indicate antenatal depression or anxiety respectively.

### 2.3 Negative controls: partner depression and anxiety

Mother’s partners completed the EPDS, validated for use in fathers (Edmondson et al., 2010), and CCEI at 18 weeks’ gestation. We used the same thresholds as above to derive binary variables for partner depression and anxiety during pregnancy.

### 2.4 Outcomes: daytime wetting, bedwetting, soiling, and constipation at age 7 years

Mothers were asked ‘how often does your child: wet themselves during the day; wet the bed at night; and dirty their pants during the day’. Options included ‘never’; ‘occasional accidents but less than once a week’; ‘about once a week’; ‘2-5 times a week’; ‘nearly every day’; and ‘more than once a day’. We derived binary variables for daytime wetting, bedwetting, and soiling (occasional and more versus never). Mothers were also asked ‘has your child had any constipation in the past 12 months’: ‘yes and saw a doctor’; ‘yes but did not see a doctor’; ‘no did not have’. We derived a binary variable indicating any constipation versus none.

### 2.5 Confounders

We adjusted for mother’s age at delivery, parity (‘1 or less’ or ‘2 or more’) and socioeconomic factors derived from responses to questionnaires during pregnancy including: occupational social class (using the 1991 British Office of Population and Census Statistics classification and dichotomised into ‘non-manual’ or ‘manual’); maternal education (‘A-level or degree’, ‘O level’, and ‘Certificate of Secondary Education/vocational qualification/none’); home ownership (‘owner or private renter’ and ‘renter or non-homeowner’); and a continuous score of financial difficulties. In models that included postnatal maternal psychopathology as the exposure, we additionally adjusted for child’s sex, gestation at birth (‘<32 weeks’, ‘32−36 weeks’, ‘37−41 weeks’, or ‘≥42 weeks’), and weight at delivery (‘<2500 g’, ‘2500−4499 g’, or ‘≥4500 g’).

### 2.6 Statistical analysis

First, we used multivariable logistic regression models to examine associations between maternal psychopathology, separately in the antenatal and postnatal periods, and offspring incontinence/constipation (N = 6489). We examined models including both depression and anxiety to test for independent effects. Next, we examined whether exposure to antenatal or postnatal maternal psychopathology was more important in determining the risk of offspring incontinence/constipation by deriving a 4-level maternal depression/anxiety variable (no antenatal or postnatal exposure; antenatal exposure only; postnatal exposure only; and exposure in both periods) and testing the fit of different life-course models to the data. We based this analysis on a life-course model approach proposed by Mishra et al. (2009) whereby a series of nested, constrained models (representing a sensitive or critical antenatal or postnatal period) were compared with an unconstrained model using likelihood ratio (LR) tests (see Supplementary materials for further details). Lastly, we examined the evidence for a causal intrauterine effect of antenatal maternal depression and anxiety on offspring incontinence/constipation using partner depression and anxiety assessed during the mother’s pregnancy as negative controls. The negative control analysis was conducted on a reduced sample (N = 5337) comprising participants with data available for both maternal and partner psychopathology assessed during pregnancy. We estimated the associations of maternal and partner depression and anxiety with offspring incontinence and constipation using multivariable logistic regression models adjusted for confounders. All analyses were conducted in Stata, version 17.

## 3 Results

The analysis focused on 6489 children with complete data on all maternal psychopathology exposures, childhood incontinence and constipation outcomes at age 7, and confounders. There were 512 (7.9 %) children with daytime wetting, 1009 (15.6 %) with bedwetting, 446 (6.9 %) with soiling, and 663 (10.2 %) with constipation.

Table 1 shows the distribution of maternal and paternal psychopathology and confounders in the samples considered for this study. The restricted samples had lower proportions of antenatal maternal depression and anxiety, manual social class, home renters/non-owners, low maternal education, mothers with two or more pregnancies and children born prematurely or of low birthweight. There was little difference in the proportion of mothers with postnatal depression and anxiety across the samples. Proportions of partner depression and anxiety differed only slightly across the samples. There was little difference in mean maternal age at delivery or mean financial difficulties score across the samples and the sex distribution remained constant.

### 3.1 Associations between maternal depression and anxiety in the antenatal and postnatal periods and offspring incontinence and constipation at age 7

The results in Table 2 provide evidence that antenatal depression and anxiety were associated with increased odds of offspring constipation at age 7 after adjustment for confounders. The association between antenatal depression and constipation was attenuated after adjustment for antenatal anxiety, but the association with antenatal anxiety remained after adjustment for antenatal depression. There was no evidence of associations between antenatal depression or anxiety and daytime wetting, bedwetting or soiling in the adjusted models.

Postnatal depression and anxiety were associated with increased odds of daytime wetting, bedwetting, soiling, and constipation in the adjusted models. The associations with postnatal depression were attenuated following adjustment for postnatal anxiety, but there was still evidence for an association with soiling. The associations remained between postnatal anxiety and all outcomes, except bedwetting, after adjustment for postnatal depression.

### 3.2 Life-course approach: testing for a critical/sensitive antenatal or postnatal period

Table 3 provides the adjusted odds ratios for the associations between the 4-level depression and anxiety exposure variables and the outcomes, and P values for the LR tests comparing the unconstrained and constrained models representing the different life-course hypotheses (Supplementary Table 1 presents the unadjusted analysis). There was evidence that the postnatal period was a critical period of exposure for maternal depression and anxiety and offspring daytime wetting, bed-wetting, and soiling, but there was no support for an antenatal critical period model. Whilst there was some support for a sensitive antenatal period model in relation to these outcomes, the effect estimates for those exposed to maternal depression/anxiety only in the postnatal period were greater than the antenatal period, which is inconsistent with the antenatal period being sensitive. For constipation, there was some support for the antenatal period being a critical period of exposure for maternal depression; the critical postnatal period model was not supported, and the sensitive postnatal period model did not yield estimates consistent with that hypothesis. There was little support for a critical/sensitive antenatal or postnatal period model when examining the relationship between maternal anxiety and offspring constipation. Instead, there were increased odds of constipation for offspring exposed to maternal anxiety irrespective of the period of exposure.

### 3.3 Negative control approach

Table 4 presents the adjusted odds ratios for the associations between maternal and partner depression and anxiety and offspring incontinence and constipation, with parents included in the same model (Supplementary Table 2 presents the unadjusted analysis and the analyses with parents included in separate models).

For constipation, there was a stronger association with maternal, compared with partner, depression. There was, however, considerable overlap in the 95 % confidence intervals which is not consistent with a causal intrauterine effect. Both mother and partner anxiety were associated with offspring constipation. There was evidence for a stronger association between partner antenatal anxiety and offspring soiling. Maternal and partner depression and anxiety during pregnancy were not associated with daytime wetting or bedwetting.

## 4 Discussion

We found evidence that exposure to maternal psychopathology in the postnatal period is associated with an increased risk of offspring incontinence and constipation at age 7. The associations with postnatal depression were attenuated following adjustment for postnatal anxiety, but the association between postnatal depression and soiling remained. There was evidence for an independent effect of maternal postnatal anxiety on offspring daytime wetting, soiling and constipation when models were adjusted for maternal postnatal depression. When we tested different life-course hypotheses, our results provided support for the postnatal, but not the antenatal, period being a critical period of exposure for maternal psychopathology in relation to offspring daytime wetting, bedwetting, and soiling. For constipation, there was some support for the antenatal period being a critical period of exposure for maternal depression. The risk of constipation in offspring exposed to maternal anxiety was increased irrespective of the period of exposure. There was little support for a causal intra-uterine effect of maternal antenatal psychopathology on offspring constipation. Instead, shared environmental or genetic factors provide a more likely explanation for the observed associations.

Previous studies have reported associations between postnatal maternal psychopathology and daytime wetting, bedwetting, soiling, and constipation (Appak et al., 2017; Joinson et al., 2019a, 2009, 2008), but did not examine if maternal depression and anxiety have independent effects. The current study found that postnatal maternal anxiety is more strongly associated with offspring incontinence than maternal depression. There is evidence that maternal anxiety and depression have different effects on offspring outcomes (Glover, 2014), possibly due to differential effects of distinct symptoms of anxiety (e.g. hyperarousal) and depression (e.g. low positive affect) on child development (Walker et al., 2020). Although some previous studies identified associations between antenatal maternal psychopathology and offspring incontinence (Joinson et al., 2019a), the current findings indicate that such associations are unlikely to be causal and reflect residual confounding from environmental and/or genetic factors.

Although the current findings are based on evidence from a large, prospective study that adjusts for a wide range of confounders identified from available empirical evidence, it is important to emphasise that the study does not provide definitive evidence of a causal effect of postnatal maternal psychopathology on offspring incontinence/constipation. This is because residual confounding is possible in all observational studies. Before coming to any conclusions about the role of maternal psychopathology in the development of offspring continence problems and constipation, further research is needed to determine whether these observed associations are causal and to identify underlying mechanisms. Moreover, it is worthwhile to highlight that incontinence and constipation are complex outcomes with numerous predisposing and maintaining factors, as well as factors that may trigger relapse, including emotional and behavioural problems, stressful life events, and family discord (Akdemir et al., 2015; Joinson et al., 2019a). Maternal psychopathology is potentially one of these factors and future research is needed to examine the interplay with other environmental risk factors. Regarding more proximal drivers for urinary incontinence (UI), a recent study also using the ALSPAC dataset explored the roles of emotional/behaviour problems and stressful life events (Warne et al., 2023). In multivariable analyses, separation anxiety symptoms were associated with new onset UI; moreover, there was evidence that females, but not males, who experienced stressful life events were at higher risk of new onset UI (Warne et al., 2023). Overall, emerging data suggest a complex interplay of multiple proximal and distal risk factors. In the context of such recent findings, exposure to maternal psychopathology during the perinatal period may play a role, but future research is needed to examine if this is a direct or indirect effect on later UI in the offspring.

There are several possible biopsychosocial mechanisms that could underlie the observed associations. Shared genes could increase the risk of psychopathology in mothers and offspring, and these genes could also increase the risk of continence problems and constipation. Maternal-offspring transmission of the gut microbiome is another possible mechanism (Ferretti et al., 2018) that could explain some of the current findings as it has been associated with both psychopathology and constipation (Avelar Rodriguez et al., 2021; Peirce and Alviña, 2019). Exposure to maternal psychopathology is associated with increased offspring stress (Ashman et al., 2002) and there is growing evidence that exposure to chronic stress affects bladder functioning (Chess-Williams et al., 2021). Several biological mechanisms have been suggested for the link between stress and bladder problems. Corticotropin-releasing factor (which is increased in response to stress) has been suggested to play a role in stress-induced urinary dysfunction (Chess-Williams et al., 2021). Afferent nerve hypersensitivity and inflammatory responses are other plausible biological mechanisms linking stress to bladder function (Chess-Williams et al., 2021). There is also a wealth of evidence concerning the effect of stress on bowel functioning through the gut-brain axis, including during the early postnatal years (Jena et al., 2020). Moreover, maternal psychopathologies in early environment may influence long-term physical and mental health outcomes in offspring through epigenetic mechanisms. A recent study identified associations between maternal attachment insecurity, maltreatment history and depressive symptoms with DNA methylation signatures in the infants (Robakis et al., 2022). Maternal psychopathology is also associated with parenting behaviours including increased hostility and less positive and warm parenting, which have been found to have adverse effects on child development (Rogers et al., 2020). Maternal psychopathology could affect caregiving behaviours around the time of toilet training, including the use of inconsistent strategies and less responsiveness to the child’s toileting needs. Unsuccessful attempts at toilet training are also associated with family conflict and stress (Taubman, 1997). There is growing evidence that inadequate toilet training practices and delayed training are associated with an increased risk of bladder and bowel disorders (Bakker and Wyndaele, 2000; Li et al., 2020; Mota and Barros, 2008). Further, maternal psychopathology is associated with emotional and behavioural problems in children (Goodman et al., 2011), which have been shown to be associated with an increased risk of continence problems and constipation (Joinson et al., 2019a, 2019b).

Major strengths of this study include the prospective design and the availability of data from a large birth cohort on validated measures of maternal depression and anxiety, and a wide range of confounders. Maternal depression and anxiety were assessed during pregnancy and postnatally, enabling us to examine whether these disorders have independent effects on offspring incontinence and constipation and allowing us to test whether exposure to maternal psychopathology in the antenatal or postnatal period was more important. Paternal negative controls enabled us to account for measured and unmeasured confounding shared between mothers and partners, although it was not possible to account for any unmeasured confounding that was unique to mothers or partners.

Potential limitations of this study are the reliance on maternal reports of their child’s incontinence and constipation, and the lack of use of established diagnostic criteria. Whilst parents are likely to be aware of their child’s urinary and faecal incontinence, it is possible that some parents were unaware of their children’s constipation, especially if accompanied by soiling. The prevalence of constipation (10.2 %) observed at age 7 in this cohort, however, is higher than the median prevalence (8.9 %) reported in a systematic review of 0-to 18-year-olds (van den Berg et al., 2006), which suggests that constipation was not underreported. We chose to focus on any level of incontinence and constipation in this community sample, rather than on children who met currently established diagnostic criteria. There is evidence that children who experience incontinence and constipation below thresholds for clinical diagnosis still experience psychological distress (Joinson et al., 2006a). Additionally, the ALSPAC dataset includes many variables which could give rise to spurious associations and thus Type 1 errors. However, it is very important to emphasise that the current work builds on previous studies in other samples that have found a relationship between maternal psychopathology and childhood incontinence. Our work is therefore embedded within a research programme with a clear set of research questions that were generated a priori through examination of the empirical literature. By presenting effect estimates with their 95 % CIs and P-values, we also avoided an arbitrary P value threshold to determine statistical significance. Our findings should therefore be interpreted with caution in the context of our broader considerations and larger corpus of existing literature. Furthermore, the demographic characteristics of ALSPAC participants limit generalisability of the findings to predominantly affluent, White UK populations. Further research is needed to determine whether the findings apply to more deprived populations and different ethnicities. The complete case sample used in this study was more socially advantaged than the original starting sample, but our estimates should be unbiased provided there are no systematic differences in the rates of the outcomes considered, after conditioning on the exposure and confounders included in the model (Hughes et al., 2019). Since our list of confounders is extensive and childhood incontinence in ALSPAC has been shown to be only weakly socially patterned (Butler and Heron, 2008), we believe this assumption is tenable in this current study.

## 5 Conclusions

The current study provides evidence that exposure to maternal psychopathology, particularly maternal anxiety, before the child’s third birthday may be associated with an increased likelihood of offspring incontinence and constipation at age 7. The findings also suggest that any associations between antenatal maternal psychopathology and offspring incontinence/constipation are more likely to be the result of environmental or genetic factors as opposed to a causal intra-uterine effect. Future research should attempt to establish whether the associations between postnatal maternal anxiety and offspring incontinence/constipation are causal. Research is also needed to identify underlying mechanisms that explain these relationships, because this knowledge could inform evidence-based support and interventions aimed at reducing the risk of bladder and bowel problems in children. Our findings suggest that primary care health professionals should be vigilant to maternal psychopathology and its effects on child development, and parents should be offered support to deal with distress.

## Supplementary Material

Supplementary Materials

## Figures and Tables

**Table 1:** Distribution of maternal and paternal psychopathology, child incontinence, and confounding factors in the samples considered for this study.

	Data on maternal antenatal EPDS & CCEI N ≤ 13,098	Data on maternal antenatal and postnatal EPDS & CCEI N ≤ 9272	Data on maternal EPDS & CCEI and continence outcomes N ≤ 7016	Data on maternal EPDS & CCEI, continence outcomes, and confounders N = 6489
**Maternal psychopathology**				
Maternal depression at 18/32 weeks gestation	1661 (12.7 %)	942 (10.2 %)	652 (9.3 %)	575 (8.9 %)
Maternal anxiety at 18/32 weeks gestation	1952 (14.9 %)	1197 (12.9 %)	837 (11.9 %)	742 (11.4 %)
Maternal depression at 33 months	1131 (12.2 %)	1128 (12.2 %)	820 (11.7 %)	745 (11.5 %)
Maternal anxiety at 33 months	1397 (15.0 %)	1393 (15.0 %)	1003 (14.3 %)	911 (14.0 %)
**Partner psychopathology**				
Partner depression at 18 weeks gestation	392 (4.1 %)	248 (3.4 %)	188 (3.3 %)	176 (3.3 %)
Partner anxiety at 18 weeks gestation	481 (5.0 %)	335 (4.6 %)	246 (4.3 %)	232 (4.3 %)
**Confounders**				
Child’s sex (male)	6728 (51.7 %)	4766 (51.4 %)	3588 (51.1 %)	3318 (51.1 %)
Manual social class (ref: non-manual)	5630 (49.0 %)	4042 (45.9 %)	2933 (43.3 %)	2796 (43.1 %)
Renter/non-homeowner (ref: homeowner/private renter)	2327 (18.6 %)	1280 (14.1 %)	804 (11.6 %)	693 (10.7 %)
Parity − 2 or more (ref: 0 or 1)	2580 (20.3 %)	1713 (18.8 %)	1228 (17.8 %)	1138 (17.5 %)
Mean financial difficulties score (SD)	2.91 (3.54)	2.68 (3.40)	2.49 (3.29)	2.47 (3.28)
Mean maternal age at delivery (SD)	28.12 (4.91)	28.73 (4.66)	29.11 (4.54)	29.14 (4.48)
Maternal education (reference category: a level/degree)				
CSE/vocational/none	3634 (30.0 %)	2276 (25.1 %)	1543 (22.3 %)	1360 (21.0 %)
O level	4196 (34.7 %)	3234 (35.6 %)	2426 (35.1 %)	2295 (35.4 %)
Gestation at birth (reference category: 37−41 weeks)				
<32 weeks	182 (1.4 %)	59 (0.6 %)	45 (0.6 %)	26 (0.4 %)
32−36 weeks	688 (5.3 %)	456 (4.9 %)	322 (4.6 %)	295 (4.6 %)
≥42 weeks	979 (7.5 %)	688 (7.4 %)	518 (7.4 %)	481 (7.4 %)
Birth weight (reference category: 2500−4499 g)				
<2500 g	720 (5.6 %)	425 (4.6 %)	300 (4.3 %)	262 (4.0 %)
≥4500 g	240 (1.9 %)	175 (1.9 %)	124 (1.8 %)	118 (1.8 %)

Abbreviations: SD, standard deviation. Maternal/paternal depression defined as an EPDS score of 13 or higher. Maternal/paternal anxiety defined as a CCEI score of 9 or higher. Child incontinence and constipation outcomes are defined as any versus no issue.

**Table 2:** Odds ratios and 95 % confidence intervals for the association between maternal depression and anxiety in the antenatal and postnatal periods and offspring daytime wetting, bedwetting, soiling, and constipation at age 7 years (N = 6489).

	Daytime wetting	Bedwetting	Soiling	Constipation
**Antenatal maternal depression**				
Unadjusted	1.15 (0.85−1.56)	1.14 (0.91−1.44)	1.39 (1.03−1.89)	1.53 (1.19−1.96)
*P value*	0.362	0.248	0.032	0.001
Adjusted for confounders^a^	1.14 (0.84−1.56)	1.05 (0.83−1.34)	1.31 (0.96−1.80)	1.46 (1.12−1.89)
*P value*	0.403	0.659	0.093	0.004
Adjusted for antenatal anxiety	1.03 (0.71−1.50)	0.99 (0.75−1.31)	1.23 (0.84−1.79)	1.18 (0.86−1.61)
*P value*	0.877	0.952	0.288	0.304
**Antenatal maternal anxiety**				
Unadjusted	1.21 (0.92−1.57)	1.24 (1.02−1.51)	1.35 (1.03−1.78)	1.61 (1.29−2.01)
*P value*	0.172	0.035	0.031	<0.001
Adjusted for confounders^a^	1.21 (0.92−1.60)	1.16 (0.94−1.43)	1.30 (0.97−1.73)	1.57 (1.25−1.98)
*P value*	0.175	0.156	0.076	<0.001
Adjusted for antenatal depression	1.19 (0.85−1.65)	1.25 (0.97−1.59)	1.21 (0.86−1.71)	1.48 (1.12−1.96)
*P value*	0.308	0.097	0.272	0.005
**Postnatal maternal depression**				
Unadjusted	1.50 (1.17−1.93)	1.42 (1.17−1.72)	1.89 (1.47−2.43)	1.39 (1.10−1.75)
*P value*	0.001	<0.001	<0.001	0.005
Adjusted for confounders^b^	1.48 (1.15−1.91)	1.37 (1.13−1.68)	1.82 (1.41−2.35)	1.33 (1.05−1.68)
*P value*	0.003	0.002	<0.001	0.017
Adjusted for postnatal anxiety	1.22 (0.88−1.69)	1.24 (0.97−1.59)	1.50 (1.08−2.09)	0.96 (0.71−1.28)
*P value*	0.225	0.093	0.016	0.774
**Postnatal maternal anxiety**				
Unadjusted	1.52 (1.21−1.92)	1.39 (1.16−1.66)	1.79 (1.41−2.26)	1.68 (1.37−2.06)
*P value*	<0.001	<0.001	<0.001	<0.001
Adjusted for confounders^b^	1.53 (1.21−1.94)	1.34 (1.11−1.61)	1.73 (1.35−2.20)	1.63 (1.33−2.01)
*P value*	<0.001	0.002	<0.001	<0.001
Adjusted for postnatal depression	1.36 (1.01−1.83)	1.23 (0.98−1.55)	1.41 (1.03−1.92)	1.72 (1.33−2.32)
*P value*	0.045	0.079	0.032	<0.001

a Antenatal depression and anxiety models adjusted for confounders including social class (parental social class; non-manual versus manual occupation), maternal education (CSE or vocational versus O level versus A level or degree), home ownership (homeowner or private renter versus renter or non-homeowner), financial difficulties score, parity (0 or 1 versus 2 or more), and maternal age at delivery.

b Postnatal depression and anxiety models adjusted for the same confounders as the antenatal models plus child sex, gestation at birth (<32 weeks versus 32 to 36 weeks versus 37 to 41 weeks versus 42 weeks or more), and weight at delivery (<2500 g versus 2500 to 4499 g versus 4500 g or more).

**Table 3:** Adjusted odds ratios and 95 % confidence intervals for the association between maternal depression and anxiety in the antenatal period only, postnatal period only, and both periods and offspring daytime wetting, bedwetting, soiling, and constipation (N = 6489).

	Daytime wetting	Bedwetting	Soiling	Constipation
**Maternal depression**				
No depression	1.0 (ref)	1.0 (ref)	1.0 (ref)	1.0 (ref)
Antenatal depression only	1.16 (0.76−1.76)	1.04 (0.76−1.42)	1.21 (0.79−1.87)	1.41 (1.00−1.98)
Postnatal depression only	1.61 (1.19−2.16)	1.46 (1.15−1.84)	1.91 (1.41−2.57)	1.25 (0.93−1.66)
Antenatal and postnatal depression	1.29 (0.83−2.01)	1.22 (0.87−1.72)	1.73 (1.13−2.66)	1.63 (1.13−2.35)
*Omnibus P value*	0.020	0.016	<0.001	0.016
*Critical antenatal model* ^a^	LR chi2(2) = 9.19 P = 0.010	LR chi2(2) = 10.00 P = 0.007	LR chi2(2) = 17.37 P ≤ 0.001	LR chi2(2) = 2.48 P = 0.289
*Sensitive antenatal model* ^b^	LR chi2(1) = 0.13 P = 0.718	LR chi2(1) = 0.54 P = 0.463	LR chi2(1) = 1.53 P = 0.216	LR chi2(1) = 0.36 P = 0.547
*Critical postnatal model* ^c^	LR chi2(2) = 1.24 P = 0.538	LR chi2(2) = 0.83 P = 0.661	LR chi2(2) = 0.87 P = 0.647	LR chi2(2) = 4.87 P = 0.088
*Sensitive postnatal model* ^d^	LR chi2(1) = 0.75 P = 0.387	LR chi2(1) = 0.77 P = 0.381	LR chi2(1) = 0.13 P = 0.714	LR chi2(1) = 1.39 P = 0.239
**Maternal anxiety**				
No anxiety	1.0 (ref)	1.0 (ref)	1.0 (ref)	1.0 (ref)
Antenatal anxiety only	1.25 (0.85−1.82)	1.16 (0.87−1.55)	1.05 (0.68−1.62)	1.69 (1.23−2.30)
Postnatal anxiety only	1.68 (1.26−2.23)	1.42 (1.13−1.78)	1.71 (1.26−2.30)	1.72 (1.33−2.23)
Antenatal and postnatal anxiety	1.37 (0.94−1.99)	1.27 (0.95−1.68)	1.78 (1.24−2.55)	1.73 (1.26−2.36)
*Omnibus P value*	0.003	0.014	<0.001	<0.001
*Critical antenatal model* ^a^	LR chi2(2) = 11.84 P = 0.003	LR chi2(2) = 8.81 P = 0.012	LR chi2(2) = 15.10 P ≤ 0.001	LR chi2(2) = 15.64 P ≤ 0.001
*Sensitive antenatal model* ^b^	LR chi2(1) = 0.14 P = 0.713	LR chi2(1) = 0.19 P = 0.666	LR chi2(1) = 3.92 P = 0.048	LR chi2(1) = 0.01 P = 0.911
*Critical postnatal model^c^*	LR chi2(2) = 2.12 P = 0.347	LR chi2(2) = 1.51 P = 0.471	LR chi2(2) = 0.06 P = 0.971	LR chi2(2) = 9.93 = 0.007
*Sensitive postnatal model^d^*	LR chi2(1) = 0.84 P = 0.359	LR chi2(1) = 0.43 P = 0.510	LR chi2(1) = 0.02 P = 0.887	LR chi2(1) = 0.00 P = 0.978

A small P value means the constrained model is not a good fit for the data compared to the unconstrained model, whereas a large P value means the constrained model fits the data as well as the unconstrained model. Models adjusted for social class (parental social class; non-manual versus manual occupation), maternal education (CSE or vocational versus O level versus A level or degree), home ownership (homeowner or private renter versus renter or non-homeowner), financial difficulties score, parity (0 or 1 versus 2 or more), maternal age at delivery, child sex, gestation at birth (<32 weeks versus 32 to 36 weeks versus 37 to 41 weeks versus 42 weeks or more), and weight at delivery (<2500 g versus 2500 to 4499 g versus 4500 g or more).

a Critical antenatal model constrains the estimate effect of ‘antenatal depression/anxiety only’ to be equal to ‘antenatal and postnatal depression/anxiety’ and the estimate of ‘postnatal depression/anxiety’ to be zero.

b Sensitive antenatal model constrains the estimate of ‘antenatal depression/anxiety only’ to be equal to ‘antenatal and postnatal depression/anxiety’.

c Critical postnatal model constrains the estimate of ‘postnatal depression/anxiety only’ to be equal to ‘antenatal and postnatal depression/anxiety’ and the estimate of ‘antenatal depression/anxiety’ to be zero.

d Sensitive postnatal model constrains the estimate of ‘postnatal depression/anxiety only’ to be equal to ‘antenatal and postnatal depression/anxiety’.

**Table 4:** Odds ratios and 95 % confidence intervals for the association between maternal and paternal depression and anxiety in the antenatal period and offspring daytime wetting, bedwetting, soiling, and constipation, adjusted for confounders and with parents included in the same model (N = 5337).

	Daytime wetting			Bedwetting			Soiling			Constipation	
	Maternal	Partner		Maternal	Partner		Maternal	Partner		Maternal	Partner
**Depression**											
Adjusted *P value*	1.20 (0.84−1.71) 0.320	0.75 (0.40−1.41) 0.373		0.90 (0.68−1.19) 0.472	1.37 (0.93−2.00) 0.108		1.26 (0.88−1.81) 0.204	1.62 (1.00−2.64) 0.052		1.54 (1.15−2.06) 0.004	1.48 (0.96−2.27) 0.074
**Anxiety**											
Adjusted *P value*	1.15 (0.84−1.58) 0.389	1.09 (0.68−1.74) 0.734		1.02 (0.80−1.29) 0.881	1.32 (0.95−1.85) 0.099		1.30 (0.95−1.78) 0.107	1.72 (1.13−2.62) 0.011		1.65 (1.28−2.12) <0.001	1.49 (1.03−2.17) 0.036

Adjusted for social class (parental social class; non-manual versus manual occupation), maternal education (CSE or vocational versus O level versus A level or degree), home ownership (homeowner or private renter versus renter or non-homeowner), financial difficulties score, parity (0 or 1 versus 2 or more), and maternal age at delivery.

## References

[R1] Akdemir D, Çengel Kültür SE, Saltik Temizel IN, Zeki A, Şenses Dinç G (2015). Familial psychological factors are associated with encopresis. Pediatr Int.

[R2] Appak YÇ, Sapmaz ŞY, Doğan G, Herdem A, Özyurt BC, Kasirga E (2017). Clinical findings, child and mother psychosocial status in functional constipation. Turk J Gastroenterol.

[R3] Ashman SB, Dawson G, Panagiotides H, Yamada E, Wilkinson CW (2002). Stress hormone levels of children of depressed mothers. Dev Psychopathol.

[R4] Avelar Rodriguez D, Popov J, Ratcliffe EM, Toro Monjaraz EM (2021). Functional constipation and the gut microbiome in children: preclinical and clinical evidence. Front Pediatr.

[R5] Bakker E, Wyndaele JJ (2000). Changes in the toilet training of children during the last 60 years: the cause of an increase in lower urinary tract dysfunction?. BJU Int.

[R6] Beck CT (1998). The effects of postpartum depression on child development: a meta-analysis. Arch Psychiatr Nurs.

[R7] Bongers ME, Tabbers MM, Benninga MA (2007). Functional nonretentive fecal incontinence in children. J Pediatr Gastroenterol Nutr.

[R8] Boyd A, Golding J, Macleod J, Lawlor DA, Fraser A, Henderson J, Molloy L, Ness A, Ring S, Smith GD (2013). Cohort profile: the ‘Children of the 90s’-the index offspring of the Avon longitudinal study of parents and children. Int J Epidemiol.

[R9] Butler RJ, Golding J, Northstone K (2005). Nocturnal enuresis at 7.5 years old: prevalence and analysis of clinical signs. BJU Int.

[R10] Butler RJ, Heron J (2008). The prevalence of infrequent bedwetting and nocturnal enuresis in childhood. Scand J Urol Nephrol.

[R11] Chess-Williams R, McDermott C, Sellers DJ, West EG, Mills KA (2021). Chronic psychological stress and lower urinary tract symptoms. LUTS: Lower Urinary Tract Symptoms.

[R12] Cox JL, Holden JM, Sagovsky R (1987). Detection of postnatal depression: development of the 10-item Edinburgh postnatal depression scale. Br J Psychiatry.

[R13] Crisp AH, Jones MG, Slater P (1978). The Middlesex hospital questionnaire: a validity study. Br J Med Psychol.

[R14] Durmaz O, Kemer S, Mutluer T, Bütün E (2017). Psychiatric dimensions in mothers of children with primary nocturnal enuresis: a controlled study. J Pediatr Urol.

[R15] Edmondson OJH, Psychogiou L, Vlachos H, Netsi E, Ramchandani PG (2010). Depression in fathers in the postnatal period: assessment of the Edinburgh postnatal depression scale as a screening measure. J Affect Disord.

[R16] Evans J, Heron J, Francomb H, Oke S, Golding J (2001). Cohort study of depressed mood during pregnancy and after childbirth. Br Med J.

[R17] Feldman R, Granat A, Pariente C, Kanety H, Kuint J, Gilboa-Schechtman E (2009). Maternal depression and anxiety across the postpartum year and infant social engagement, fear regulation, and stress reactivity. J Am Acad Child Adolesc Psychiatry.

[R18] Ferretti P, Pasolli E, Tett A, Asnicar F, Gorfer V, Fedi S, Armanini F, Truong DT, Manara S, Zolfo M, Beghini F (2018). Mother-to-infant microbial transmission from different body sites shapes the developing infant gut microbiome. Cell Host Microbe.

[R19] Fraser A, Macdonald-wallis C, Tilling K, Boyd A, Golding J, Davey Smith G, Henderson J, Macleod J, Molloy L, Ness A, Ring S (2013). Cohort profile: the avon longitudinal study of parents and children: ALSPAC mothers cohort. Int J Epidemiol.

[R20] Glover V (2014). Maternal depression, anxiety and stress during pregnancy and child outcome; what needs to be done. Best Pract Res Clin Obstet Gynaecol.

[R21] Goodman SH, Rouse MH, Connell AM, Broth MR, Hall CM, Heyward D (2011). Maternal depression and child psychopathology: a meta-analytic review. Clin Child Fam Psychol Rev.

[R22] Grzeda MT, Heron J, von Gontard A, Joinson C (2017). Effects of urinary incontinence on psychosocial outcomes in adolescence. Eur Child Adolesc Psychiatry.

[R23] Heron J, Grzeda MT, von Gontard A, Wright A, Joinson C (2017). Trajectories of urinary incontinence in childhood and bladder and bowel symptoms in adolescence: prospective cohort study. BMJ Open.

[R24] Hughes RA, Heron J, Sterne JAC, Tilling K (2019). Accounting for missing data in statistical analyses: multiple imputation is not always the answer. Int J Epidemiol.

[R25] Jena A, Montoya CA, Mullaney JA, Dilger RN, Young W, McNabb WC, Roy NC (2020). Gut-brain axis in the early postnatal years of life: a developmental perspective. Front Integr Neurosci.

[R26] Joinson C, Grzeda MT, von Gontard A, Heron J (2019a). A prospective cohort study of biopsychosocial factors associated with childhood urinary incontinence. Eur Child Adolesc Psychiatry.

[R27] Joinson C, Grzeda MT, von Gontard A, Heron J (2019b). Psychosocial risks for constipation and soiling in primary school children. Eur Child Adolesc Psychiatry.

[R28] Joinson C, Heron J, Butler R, Croudace T (2009). Development of nighttime bladder control from 4−9 years: association with dimensions of parent rated child maturational level, child temperament and maternal psychopathology. Longit Life Course Stud.

[R29] Joinson C, Heron J, Butler U, von Gontard A (2006a). Psychological differences between children with and without soiling problems. Pediatrics.

[R30] Joinson C, Heron J, von Gontard A (2006b). Psychological problems in children with daytime wetting. Pediatrics.

[R31] Joinson C, Heron J, von Gontard A, Butler U, Golding J, Emond A (2008). Early childhood risk factors associated with daytime wetting and soiling in school-age children. J Pediatr Psychol.

[R32] Kessel EM, Allmann AES, Goldstein BL, Finsaas M, Dougherty LR, Bufferd SJ, Carlson GA, Klein DN (2017). Predictors and outcomes of childhood primary enuresis. J Am Acad Child Adolesc Psychiatry.

[R33] Li X, Wen JG, Xie H, Wu XD, Shen T, Yang XQ, Wang XZ, Chen GX, Yang MF, Du YK (2020). Delayed in toilet training association with pediatric lower urinary tract dysfunction: a systematic review and meta-analysis. J Pediatr Urol.

[R34] Lipsitch M, Tchetgen Tchetgen E, Cohen T (2010). Negative controls: a tool for detecting confounding and bias in observational studies. Epidemiology.

[R35] Mishra G, Nitsch D, Black S, de Stavola B, Kuh D, Hardy R (2009). A structured approach to modelling the effects of binary exposure variables over the life course. Int J Epidemiol.

[R36] Moss KM, Dobson AJ, Mishra GD (2020). Testing the role of the timing and chronicity of maternal depressive symptoms in the associations with child behaviour and development. Paediatr Perinat Epidemiol.

[R37] Mota DM, Barros AJD (2008). Toilet training: methods, parental expectations and associated dysfunctions. J Pediatr.

[R38] Naitoh Y, Kawauchi A, Soh J, Kamoi K, Miki T (2012). Health related quality of life for monosymptomatic enuretic children and their mothers. J Urol.

[R39] National Research Council (2009). Depression in Parents, Parenting, and Children: Opportunities to Improve Identification, Treatment, and Prevention.

[R40] Peirce JM, Alviña K (2019). The role of inflammation and the gut microbiome in depression and anxiety. J Neurosci Res.

[R41] Rahman A, Iqbal Z, Bunn J, Lovel H, Harrington R (2004). Impact of maternal depression on infant nutritional status and illness. Arch Gen Psychiatry.

[R42] Raposa E, Hammen C, Brennan P, Najman J (2014). The long-term effects of maternal depression: early childhood physical health as a pathway to offspring depression. J Adolesc Health.

[R43] Robakis TK, Roth MC, King LS, Humphreys KL, Ho M, Zhang X, Chen Y, Li T, Rasgon NL, Watson KT, Urban AE (2022). Maternal attachment insecurity, maltreatment history, and depressive symptoms are associated with broad DNA methylation signatures in infants. Mol Psychiatry.

[R44] Rogers A, Obst S, Teague SJ, Rossen L, Spry EA, MacDonald JA, Sunderland M, Olsson CA, Youssef G, Hutchinson D (2020). Association between maternal perinatal depression and anxiety and child and adolescent development: a meta-analysis. JAMA Pediatr.

[R45] Taubman B (1997). Toilet training and toileting refusal for stool only: a prospective study. Pediatrics.

[R46] Thibodeau BA, Metcalfe P, Koop P, Moore K (2013). Urinary incontinence and quality of life in children. J Pediatr Urol.

[R47] van den Berg MM, Benninga MA, di Lorenzo C (2006). Epidemiology of childhood constipation: a systematic review. Am J Gastroenterol.

[R48] Walker AL, Peters PH, de Rooij SR, Henrichs J, Witteveen AB, Verhoeven CJM, Vrijkotte TGM, de Jonge A (2020). The long-term impact of maternal anxiety and depression postpartum and in early childhood on child and paternal mental health at 11−12 years follow-up. Front Psychiatry.

[R49] Warne N, Heron J, von Gontard A, Joinson C (2023). Mental health problems, stressful life events and new-onset urinary incontinence in primary school-age children: a prospective cohort study. Eur Child Adolesc Psychiatry.

